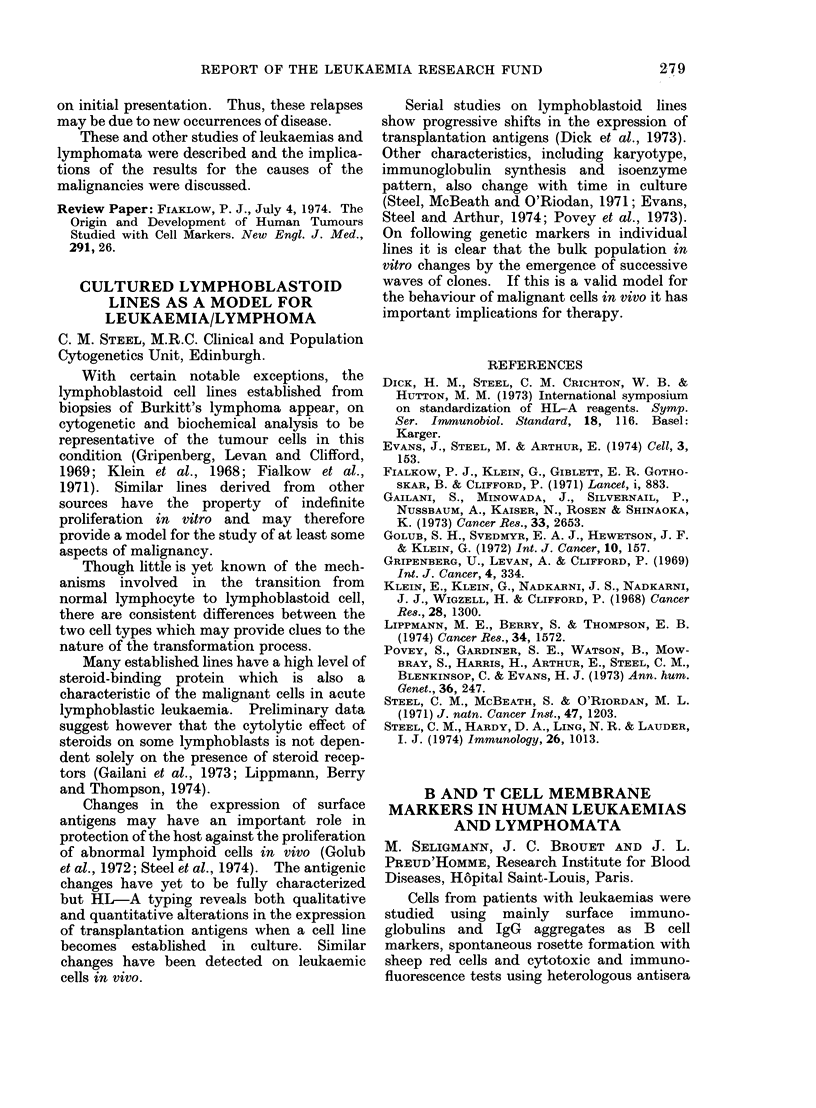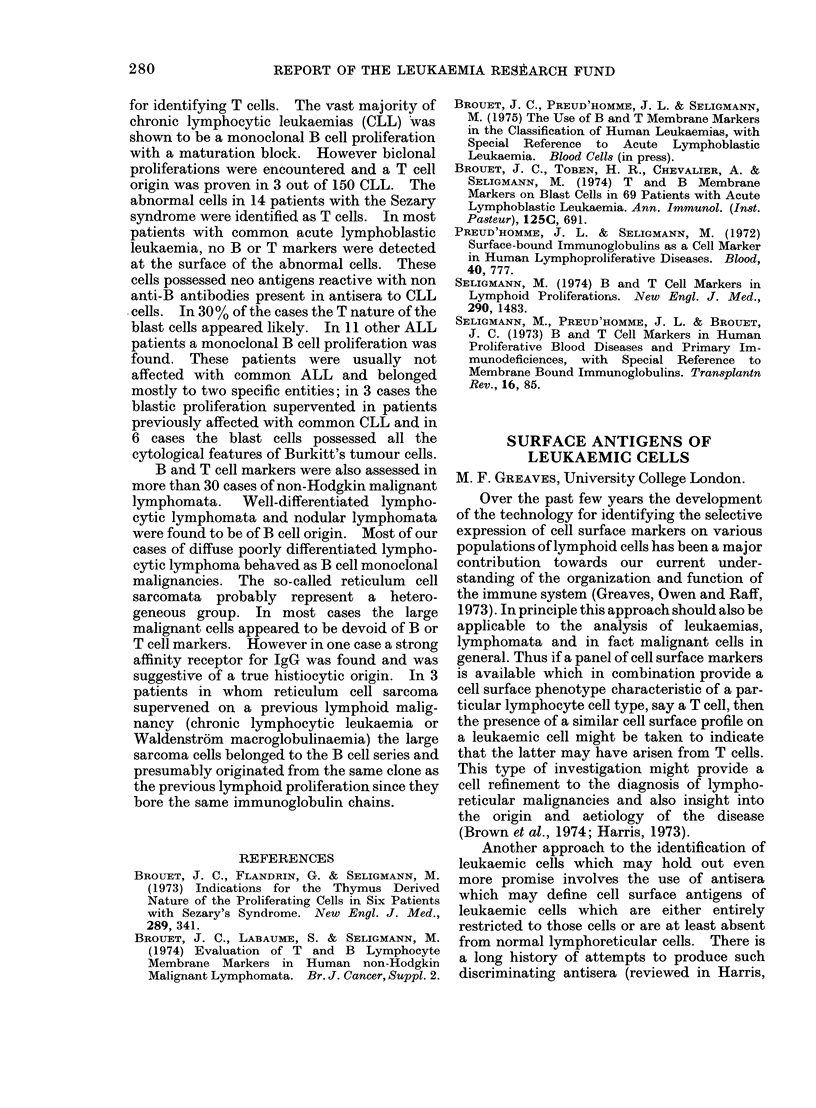# Proceedings: B and T cell membrane markers in human leukaemias and lymphomata.

**DOI:** 10.1038/bjc.1975.217

**Published:** 1975-08

**Authors:** M. Seligmann, J. C. Brouet, J. L. Preud'Homme


### B AND T CELL MEMBRANE MARKERS IN HUMAN LEUKAEMIAS AND LYMPHOMATA

M. SELIGMANN, J. C. BROUET AND J. L. PREUD'HOMME, Research Institute for Blood Diseases, Hôpital Saint-Louis, Paris.

Cells from patients with leukaemias were studied using mainly surface immunoglobulins and IgG aggregates as B cell markers, spontaneous rosette formation with sheep red cells and cytotoxic and immunofluorescence tests using heterologous antisera for identifying T cells. The vast majority of chronic lymphocytic leukaemias (CLL) was shown to be a monoclonal B cell proliferation with a maturation block. However biclonal proliferations were encountered and a T cell origin was proven in 3 out of 150 CLL. The abnormal cells in 14 patients with the Sezary syndrome were identified as T cells. In most patients with common acute lymphoblastic leukaemia, no B or T markers were detected at the surface of the abnormal cells. These cells possessed neo antigens reactive with non anti-B antibodies present in antisera to CLL cells. In 30% of the cases the T nature of the blast cells appeared likely. In 11 other ALL patients a monoclonal B cell proliferation was found. These patients were usually not affected with common ALL and belonged mostly to two specific entities; in 3 cases the blastic proliferation supervented in patients previously affected with common CLL and in 6 cases the blast cells possessed all the cytological features of Burkitt's tumour cells.

B and T cell markers were also assessed in more than 30 cases of non-Hodgkin malignant lymphomata. Well-differentiated lymphocytic lymphomata and nodular lymphomata were found to be of B cell origin. Most of our cases of diffuse poorly differentiated lymphocytic lymphoma behaved as B cell monoclonal malignancies. The so-called reticulum cell sarcomata probably represent a heterogeneous group. In most cases the large malignant cells appeared to be devoid of B or T cell markers. However in one case a strong affinity receptor for IgG was found and was suggestive of a true histiocytic origin. In 3 patients in whom reticulum cell sarcoma supervened on a previous lymphoid malignancy (chronic lymphocytic leukaemia or Waldenström macroglobulinaemia) the large sarcoma cells belonged to the B cell series and presumably originated from the same clone as the previous lymphoid proliferation since they bore the same immunoglobulin chains.

REFERENCES

Brouet, J. C., Flandrin, G. & Seligmann, M. (1973) Indications for the Thymus Derived Nature of the Proliferating Cells in Six Patients with Sezary's Syndrome. *New Engl. J. Med.*, **289**, 341.

Brouet, J. C., Labaume, S. & Seligmann, M. (1974) Evaluation of T and B Lymphocyte Membrane Markers in Human non-Hodgkin Malignant Lymphomata. *Br. J. Cancer, Suppl.* 2.

Brouet, J. C., Preud'homme, J. L. & Seligmann, M. (1975) The Use of B and T Membrane Markers in the Classification of Human Leukaemias, with Special Reference to Acute Lymphoblastic Leukaemia. *Blood Cells* (in press).

Brouet, J. C., Toben, H. R., Chevalier, A. & Seligmann, M. (1974) T and B Membrane Markers on Blast Cells in 69 Patients with Acute Lymphoblastic Leukaemia. *Ann. Immunol.* (*Inst. Pasteur*), **125C**, 691.

Preud'homme, J. L. & Seligmann, M. (1972) Surface-bound Immunoglobulins as a Cell Marker in Human Lymphoproliferative Diseases. *Blood*, **40**, 777.

Seligmann, M. (1974) B and T Cell Markers in Lymphoid Proliferations. *New Engl. J. Med.*, **290**, 1483.

Seligmann, M., Preud'homme, J. L. & Brouet, J. C. (1973) B and T Cell Markers in Human Proliferative Blood Diseases and Primary Immunodeficiences, with Special Reference to Membrane Bound Immunoglobulins. *Transplantn Rev.*, **16**, 85.